# Narcissistic leadership, workplace bullying, turnover intention, and creative performance: a study of nurses

**DOI:** 10.1186/s12912-025-03479-x

**Published:** 2025-07-10

**Authors:** Dalia Khalid Faeq

**Affiliations:** Department of Business Administration, Faculty of Humanities and Social Science, Koya University, Koya, Kurdistan Region Iraq

**Keywords:** Narcissistic leadership, Workplace bullying, Turnover intention, Creative performance, Nurses

## Abstract

**Purpose:**

This study aims to develop and propose a research model that investigates the effects of a narcissistic leadership style on nurses. In brief, this empirical study investigates three main aims: (a) direct effect of narcissistic leadership on workplace bullying, nurses turnover intention, and creative performance; (b) effect of bullying on turnover intention and creative performance; and (c) how bullying acts as a mediator between these three variables.

**Design/methodology/approach:**

The study is a quantitative survey depending on questionnaire, the date were collected from nurses in the private hospitals in Sulaimania city, Kurdistan region of Iraq.

**Findings:**

The findings from structural equation modeling indicate that workplace bullying acts as a key mechanism linking narcissistic leadership to nurses’ turnover intention and reduced creative performance.

**Originality/value:**

No empirical study has examined the linkage between narcissistic leadership, workplace bullying, nurses turnover intention and creative performance so far. Moreover, there are few empirical studies in the current literature have tested the mechanism through which workplace bullying is associated with nurses turnover intention and creative performance.

## Introduction

Nurses in the healthcare industry are a vital asset, constituting the service capacity of health organizations and playing a critical role in enabling these institutions to operate efficiently [1] demonstrating innovative ideas and appropriate performance when needed. This can be regarded as a creative performance, which relates to the amount of innovative ideas produced and original behaviors exhibited by employees [2]. Nonetheless, the healthcare sector is experiencing significant nurses turnover intention. This results in diminished efficiency of health service provision, obstructing rural residents’ access to quality and safe healthcare services [1]. There are a number of factors that can affect an nurse’s decision to leave their current position, including: (a) demographic characteristics such as age, gender, and marital status; educational level (b) internal aspects such as management style, pay rate, benefits, and other organizational policies; and (c) external factors such as political and social instability, healthcare funding gaps, and ongoing conflicts. Replacing nurses in the healthcare sector is difficult due to the significant time and costs associated with recruitment, selection, hiring, and training, given that these individuals are highly skilled professionals [3]. Human resources are unique among organizational resources since they are neither owned nor controlled by the organization in its entirety [4]. Investment in human resources and the implementation of various strategies to eradicate negative leadership do not guarantee the retention of proficient personnel [5]. Thus, new insights could be added to the existing retention literature by looking at leadership styles from the perspective of nurses. In accordance with this reasoning, we concentrate on narcissistic leadership styles.

According to Rossenthal and Pittinsky [6] “principally motivated by the leader’s own ego-maniacal needs and beliefs, superseding the needs and interests of the constituents and institutions they lead” describes narcissistic leadership, an undesirable style of leadership. This Concentrate primarily on accomplishments, an ambition for dominance, a quest for power, and inclinations toward inflated cognitive frameworks [7].

Narcissistic leadership is distinct from other forms of unfavorable leadership. According to Aboramadan et al. [7] narcissistic leaders typically pointed towards a reluctance to accept criticism, exhibit arrogance, lack empathy, and engage in manipulative behaviors. Narcissistic leadership seems to more effectively elucidate numerous adverse outcomes (e.g., work deviance; diminished job embeddedness; reduced job engagement; lack of innovative behavior) [8–10]. Narcissistic leadership, as a detrimental leadership style, manifests in diverse environments including hospitality, banking, and the public sector [7, 11, 12].

individuals who recognize pronounced indicators of narcissistic leadership in the workplace engage in workplace bullying; bullying is intrinsic to narcissistic behavior Glad [13] and is defined as “harassing, offending, socially excluding someone, or negatively affecting someone’s work” [14]. Workplace bullying produces adverse effects, including increased individual turnover intention, diminished job satisfaction, reduced work engagement, and decreased job performance [15–18]. Narcissistic leadership incites workplace bullying, resulting in adverse consequences [19–21].

Nurse exposed to narcissistic leadership frequently experience increased work-related stress, potentially resulting in intentions to leave the organization. Moreover, such individuals not only undermine the morale of their colleagues but also obstruct the efficacy and efficiency of the service delivery process, including creative performance [22].

### Contribution

The current study aims to address multiple gaps in the healthcare literature. Initially, upon examining the literature on narcissistic leadership, we identified a paucity of studies clarifying the consequences of such leadership, exemplified by the research conducted by Erkutlu and Chafra [23] which investigated the correlation between narcissistic leaders and followers’ embeddedness in Turkish hotels. The research conducted by Aboramadan et al. [7] examined the relationship between narcissistic leadership and behavioral cynicism within the Italian hotel sector, while Norouzinik et al. [9] investigated the association between narcissistic leadership and innovative behavior. Secondly, a review of the existing literature reveals a paucity of research regarding the mechanisms by which narcissistic leadership influences significant organizational outcomes. Yao et al. [24] highlight the scarcity of empirical studies investigating the effects of narcissistic leadership on job-related outcomes. Ghislieri et al. [25] examine the inadequate empirical research regarding the mediating variables that affect the relationship between narcissistic leadership and workplace outcomes. Labrague et al. [37] similarly highlight the gap in understanding the mechanism by which narcissistic leadership affects nurses’ attitudinal and behavioral outcomes. Norouzinik [9] emphasizes the necessity for a deeper comprehension of the mediating mechanism that connects narcissistic leadership to adverse outcomes. The current service research highlights the aforementioned gap [41]. Third, the majority of empirical studies in the existing literature have predominantly concentrated on investigating narcissistic leadership in developed nations [7]. However, the examination of these practices in developing countries or emerging economies is significant due to potential variations in cultural contexts and psychological structures [25]. The study uniquely centers on the Kurdistan region of Iraq, characterized by an emerging and unstable market economy and political environment. Remarkably, this region has been underrepresented in empirical studies concerning nurses’ perceptions of narcissistic leadership styles. Consequently, this study utilizes data collected from nurses in private hospitals in Sulaimania city, Kurdistan region of Iraq.

The objective of this study is to formulate and propose a research model that investigates the effects of narcissistic leadership style on nurses within the healthcare sector, thereby addressing the identified gap in the existing literature. This empirical study examines: (a) the effects of narcissistic leadership on workplace bullying, nurses turnover intention, and creative performance; (b) the impact of workplace bullying on turnover intention and creative performance; and (c) the role of workplace bullying as a mediator in these relationships.To establish the previously mentioned relationships, our research employs both the Conservation of Resources (COR) and Job Demands-Resources (JDR) frameworks [14]. This study makes several important theoretical contributions. First, it extends the COR theory by positioning narcissistic leadership as a key stressor that depletes nurses’ emotional and psychological resources, thereby influencing their behavioral and attitudinal outcomes. Second, it contributes to the JD-R model by illustrating how narcissistic leadership and workplace bullying function as interconnected job demands that impair employees’ well-being and hinder creative output. By highlighting workplace bullying as a mediating mechanism, the study sheds light on how toxic leadership indirectly shapes critical work outcomes. Third, while previous studies have largely focused on organizational identification and engagement in leadership research, this study emphasizes the neglected yet critical role of workplace bullying in mediating the effects of narcissistic leadership, especially in healthcare contexts. Finally, the study advances the theoretical discourse by offering empirical evidence from a developing economy—Kurdistan region of Iraq—where the cultural and contextual dimensions of leadership remain underexplored. This context-sensitive approach provides new insights into the cross-cultural applicability of leadership theories and the universal relevance of COR and JD-R frameworks. The ensuing section elucidates an exploration of the aforementioned theories earmarked for incorporation in the formulation of hypotheses. Subsequently, the hypotheses and the research model are explained in alignment with the theoretical underpinnings of our study. Following this, a comprehensive discourse on the methodology employed and the resultant empirical findings obtained from our study involving employees is presented. The denouement of our study encompasses implications for both theoretical frameworks and practical applications.

## Hypotheses, and research model

An examination of current service research indicates that personality variables and leadership styles affect individuals’ attitudes and behaviors. For example, Research by Alessandri et al. [26] stated that psychological capital affected work engagement and job performance. Saeed [27] reported the mediating effect of personality dimensions on turnover intention through the psychological contract. The study by Karatepe et al. [28] established that servant leadership and organizational trust forecasted employees’ intentions to be absent, their creative performance, and their service recovery performance. Khan et al., [29] investigated the influence of transformational leadership on job performance, burnout, and social loafing. Freire and Bettencourt [30] indicated that ethical leadership influences job satisfaction through work-family conflict. Durrah and Kahwaji’s [31] recent study stated that chameleon leadership influences the innovative behavior of personnel in the healthcare sector via job security. Muzee [32] discovered that strategic leadership influenced employee engagement. The results of the aforementioned research efforts have significantly improved our understanding of the factors that affect essential job-related outcomes. This study examines the mediating mechanism connecting narcissistic leadership to turnover intention and creative performance, due to the lack of empirical evidence regarding these outcomes in contemporary service research. The COR theory facilitates the establishment of the connection between narcissistic leadership and workplace bullying. This theory defines resources as “objects, personal characteristics, conditions, or energies valued by the individual or that facilitate the acquisition of these objects, personal characteristics, conditions, or energies” [33]. Individuals strive to acquire and accumulate their limited resources. However, the presence of negative leadership or the prevalence of stressors in the workplace can lead to resource exhaustion and adverse outcomes [34]. Workplace stress arises when individuals (a) confront the potential loss of resources, (b) experience resource depletion, and (c) allocate resources without achieving the anticipated returns [35].

While this study finds that narcissistic leadership in Kurdistan’s private hospitals is positively associated with workplace bullying, increased turnover intention, and reduced creative performance, similar outcomes have been documented in developed economies such as the United States, the United Kingdom, and Germany, where narcissistic leaders have been linked to toxic workplace behaviors, employee disengagement, and decreased innovation [49]. For instance, studies in the U.S. healthcare sector have shown that narcissistic leadership correlates with higher staff burnout and turnover, while research in the UK has highlighted its negative effect on team collaboration and creativity—indicating that the detrimental impact of narcissistic leadership transcends regional and economic boundaries [51]. Individuals when exist with narcissistic leadership as a form of destructive resources and stress generator [7]. Exhibiting a pessimistic outlook and experiencing aversive mood states tend to manifest heightened reactivity in response to negative behaviors. Leading to work-related strain, such as workplace bullying.

This study extends the COR model by demonstrating how narcissistic leadership acts as a resource-draining factor that not only depletes nurses’ emotional and psychological resources but also triggers defensive responses such as turnover intention and reduced creative performance [60]. Additionally, it advances the JD-R model by positioning narcissistic leadership and workplace bullying as interrelated job demands that jointly contribute to employee strain, thereby offering a more integrated understanding of how toxic leadership styles undermine both well-being and innovation in high-pressure healthcare environments [36]. However, it appears that only one empirical study has examined the impact of narcissistic leadership on workplace bullying so far [36]. Therefore, we propose:

### H1: Narcissistic leadership is positively related to workplace bullying

Narcissistic leadership is positively related to workplace bullying. Subordinate nurses working under narcissistic supervisors tend to suffer substantial losses, both professionally and personally. This loss of resources may involve valuable work opportunities such as training, career progression, empowerment, and promotion and also personal resources like time, energy, and health. Hence, such individuals often end up resource deprived within the organization [37].

According to the Conservation of Resources (COR) theory, individuals who experience resource depletion may adopt self-defense strategies to preserve their remaining resources [35]. Turnover intention is one form of self-defensive strategy that an individual may resort to. Along the same lines, COR theory asserts that less experienced nurses with fewer resources will be less able to obtain additional resources [38]. Therefore, subjects operating under a narcissistic leadership style may be more likely to exhibit low levels of creative performance because of resource buffers making it difficult to resource richer [37].

Prior studies confirm that nurses perform best when provided with sufficient resources [37]; their absence limits current output and stifles future innovation. According to the previous research, nurses perform best when they are provided necessary resources [37]. Hence, an absence of resources not only limits the output that can be achieved now, but also stifles innovation and growth in the future.

Previous works disclosed that narsisitic leaders positively effected turnover intention [39–41], negatively impacted performance [2, 42, 43]. Accordingly, it is hypothesized that:

### H2: Narcissistic leadership (a) is positively related to turnover intention (b) and negatively related to creative performance

Adverse work-related outcomes for nurses arise from both strain and the exhaustion of valued resources [44]. When nurses encounter bullying in the workplace, they become drained of energy and emotional resources. Nurses in a detrimental work environment suggested increased turnover intention and diminished creative performance. It is argued that when nurses lack sufficient support to manage bullying, they are less likely to generate innovative ideas or maintain creative performance. Our study posits that nurses perceptions of workplace bullying result in adverse outcomes. This is unsurprising, as elevated bullying levels lead nurses to perceive their personal resources as diminished. Workplace bullying results in increased turnover intentions and diminished creative performance. A review of the relevant research reveals limited proof regarding the relationship between the aforementioned constructs. The Anasori et al. [45] study reported a significant positive correlation between workplace bullying and employee creativity. Holm et al. [17] established that workplace bullying increases turnover intention within the healthcare sector. Baek and Lee [46] found a positive correlation between workplace bullying and turnover intention among nurses in Korea in their study. De Clercq et al. [47] discovered that workplace bullying surpassed turnover intentions.Hence, it is postulated that:

### H3: Workplace bullying (a) is positively related to turnover intention (b) and negatively related to creative performance

Nurses who are facing challenges in managing and coping with stress manifest undesirable outcomes [48]. Especially with narcissistic leaders. Resources (JD-R) model, individuals exposed to high job stress—such as toxic leadership or excessive workload—experience emotional and physical resource depletion, increasing strain and workplace bullying [48]. Drawing on the health impairment component of this model, it can be posited that narcissistic leadership, identified as a job stressor, contributes to the depletion of nurses’ energy and emotional and physical resources, consequently escalating the occurrence of workplace bullying as a manifestation of work-related strain [48]. Consequently, nurses subjected to such conditions are more likely to indicate increased turnover intention and diminished creative performance as adverse outcomes.

In empirical terms, Olsen et al. [49] reported that workplace bullying intensified work-related strain, thus decreasing employees’ job performance, job satisfaction, and workability. Hence, it is postulated that:

### H4: Workplace bullying will mediate the influence of narcissistic leadership on employees’ (a) turnover intention and (b) creative performance

### Research model

**Fig. 1:**
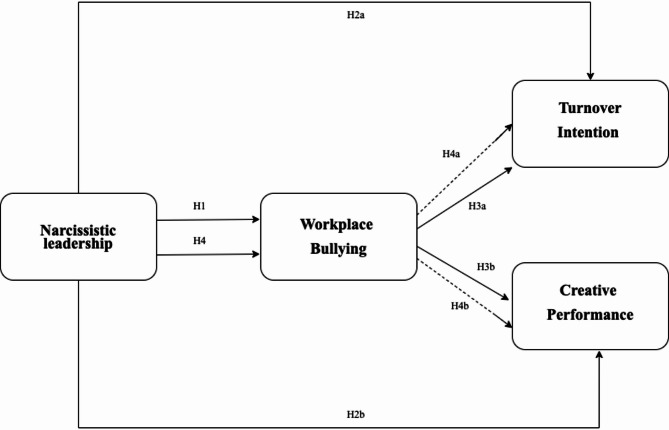
Research model

The relationships established according to COR theory and the Job Demands-Resources (JD-R) model are illustrated in the research model presented in Fig. 1. Bullying in the workplace functions as a more immediate factor within the framework of narcissistic leadership. This model posits that workplace bullying exacerbates nurses’ intentions to resign while concurrently impairing their creative performance capabilities. These connections suggest that workplace bullying mediates the impact of narcissistic leadership on the specified outcomes (Fig. 1).

## Methodology

### Sample and procedure

Our study sample comprised nurse in a private hospital located in Sulaimania city, within the Kurdistan Region of Iraq. According to information provided by the Ministry of Health in the Kurdistan Region of Iraq, there were a total of 14 private hospitals operating in Sulaimania city. Our research team initiated contact with the management of each of these hospitals through official correspondence or telephone communication. The study employed a random sampling method to collect data, ensuring that each participant had an equal chance of selection, thereby enhancing the representativeness and reducing sampling bias in the findings. The purpose of this outreach was to elucidate the study’s objectives and secure formal permission for the collection of data. Subsequently, the management of nine hospitals granted their consent for the data collection process. To minimize selection bias and enhance the representativeness of the sample, this study employed a random sampling method across private hospitals in Sulaimania city, Kurdistan Region of Iraq. By contacting all 14 private hospitals and obtaining approval from nine, the researchers aimed to ensure broad institutional participation. Each nurse in the participating hospitals was given an equal chance to be selected, and standardized procedures were followed for questionnaire distribution and collection. Nevertheless, the voluntary nature of participation may still introduce an element of selection bias, as nurses who chose to respond might systematically differ in experience or perspectives from those who declined. Furthermore, since the sample is limited to private hospitals within a single urban center, it may not fully represent the experiences of nurses in public hospitals or rural areas, which affects the generalizability of the findings. Future research should consider expanding the sampling frame to include diverse geographic and institutional contexts.

The research team simply conveyed the study’s objectives to the staff. The cover page of the questionnaire contained the following assurance: “There are no absolute right or wrong answers in this survey.” “All data gathered during our research will be maintained in strict confidentiality.” “Participation is optional, yet highly recommended,” and “the management of your hospital firmly supports and endorses your involvement.”

Each nurse received the questionnaire in a sealed envelope and was courteously asked to return the completed questionnaire in a sealed envelope, either by placing it in a plastic folder or directly handing it to a member of the research team. The majority of nurses completed the questionnaires during their breaks, while the others did so during their work shifts. Four hundred thirty-five nurses from nine private hospitals in Sulaimania, Kurdistan region, Iraq, were invited to participate in the study. Three hundred seventy-four questionnaires were submitted. As a result, questionnaires were collected, resulting in a response rate of 79.7%. Table 1 displays the profiles of the respondents.

The questionnaires were initially composed in English and later translated into Kurdish utilizing the established back-translation technique. The questionnaires were subjected to a pretesting phase with five nurses from the participating hospitals. The results of this pretest indicated no problems with the clarity and comprehensibility of the questionnaire items. Table 1 presents the respondent’s profiles.

**Table 1:** Respondents profile (*n* = 374)

	Frequency	%
Age		
18–29	97	25.93
30–39	200	53.47
40–49	30	8.02
50–59	47	12.56
**Total**	**374**	**100**
Gender		
Male	224	59.89
Female	150	40.11
**Total**	**374**	**100**
Education		
Secondary and high school	142	37.96
Diploma	70	18.72
Four-year college degree	160	42.78
Graduate degree	2	0.53
**Total**	**374**	**100**
Organization tenure		
Less than 1 year	46	12.3
1–5	88	23.53
6–10	154	41.17
11–15	26	6.95
16–20	40	10.7
More than 20	20	5.34
**Total**	**374**	**100**
Marital status		
Single or divorced	210	56.15
Married	164	43.85
**Total**	**374**	**100**

Note *N* = 374

### Narcissistic leadership

Narcissistic leadership was assessed utilizing a six-item scale created by Hochwarter and Thompson [50]. An example statement is, “My leader is exceedingly egocentric.” This study assessed narcissistic leadership utilizing a five-point Likert scale (1 = complete disagreement to 5 = complete agreement). Recent research utilized the aforementioned scale to assess servant leadership [7].

### Workplace bullying

A nine-item scale created by Einarsen et al. [51] was utilized to assess workplace bullying. The measurement instrument Examples of such items encompass “experiencing humiliation or ridicule related to your work” and “an individual withholding information that impacts your performance.” The response options comprised a five-point scale, with anchors at 1 (completely inconsistent) and 5 (completely consistent), where 5 indicated strong agreement and 1 indicated strong disagreement. Research utilized the previously mentioned scale to assess workplace bullying (52, 53).

### Turnover intention

Three items have been taken from Singh et al. [54]. The items were evaluated on a scale from 5 (strongly agree) to 1 (strongly disagree). A sample item is ‘I frequently contemplate resigning.’ Recent research employed the aforementioned three-item scale to assess lateness attitude [37].

### Creative performance

Six items were altered from Wang and Netemeyer [55] to assess creative performance (the manager questionnaire). Managers evaluated FBEs’ creative performance using a scale ranging from 5 (almost always) to 1 (never). One example item is ‘This employee performs routine tasks in a resourceful manner.’ These items were employed in recent research (e.g.,[56]).

### Data analysis

For the purpose of this study, we used SPSS 22.0 to examine the alpha coefficient, correlations between the variables, summary statistics, and frequencies. According to Anderson and Gerbing [57] AMOS is a two-step process that our study used to test the measurement and structural models. To evaluate the structural and measurement models, we used maximum likelihood estimation. To begin, we used AMOS 22.0’s confirmatory factor analysis to examine the measurement model for convergent and discriminant validity, internal consistency reliability (i.e., composite reliability), and other reliability-related properties [58, 59].

The second step was to use structural equation modeling to check the models’ relationships. The bias-corrected (BC) bootstrapping method was used to evaluate the mediating effects. Using 5000 bootstrapped samples, we estimated a 95% BC CI in our study.

A minimum of 113 participants was suggested for structural equation modeling with four latent variables (narcissistic leadership, workplace bullying, creative performance, and turnover intention), an expected effect size of 0.5, and a desired statistical power level of 0.8. A total of twenty-four variables have been recorded. Accordingly, the present study’s sample size was considered adequate [60]. The incremental fit index (IFI), Tucker-Lewis index (TLI), comparative fit index (CFI), standardized root mean square residual (SRMR), and root mean square error of approximation (RMSEA; 90% CI) were the fit statistics that were utilized for evaluating the measurement and structural models.

### Findings

#### Measurement model check

All items with standardized loadings greater than 0.50 were highlighted in the initial analysis. Here are the items from the confirmatory factor analysis: Table 2. With an overall model fit of 0.983 (CFI), 0.85 (PNFI), and 0.060 (RMSEA), the measurement model was supported (X2 = 359, df = 137, X2 = 2.62). The AVE for narcissistic leadership was 0.68, for workplace bullying it was 0.56, for turnover intention it was 0.78, and for creative performance it was 0.66, all of which were greater than 0.50, as shown in Table 2. It was clear from these results that convergent validity had been attained (see, for example, [59]). The method proposed by Fornell and Larcker [59] was used to assess discriminant validity. These values are known as the AVE. With the exception of narcissistic leadership (0.794), workplace bullying (0.89), turnover intention (0.78), and creative performance (0.88), all Cronbach’s alphas were higher than. 0.60 was also the composite reliability. According to Bagozzi and Yi [58] these results indicated that the assessments were accurate. Summary statistics and correlations of observed variables are presented in Table 2.

**Table 2:** Scale items, standardized loadings, and confirmatory factor analysis

Scale items	Standardized loading	t- value
**Narcissistic leadership** (AVE = 0.68, CR = 0.56, α = 0.794)		
Narcissistic 1	0.78	8.346
Narcissistic 2	0.78	8.514
Narcissistic 3	0.654	Fixed
Narcissistic 4	0.68	7.89
Narcissistic 5	0.68	7.918
Narcissistic 6	0.629	5.491
**Workplace Bullying** (AVE = 0.56, CR = 0.52, α = 0.89)		
Workplace Bullying 1	0.68	Fixed
Workplace Bullying 2	0.59	8.689
Workplace Bullying 3	0.78	8.944
Workplace Bullying 4	0.74	10.69
Workplace Bullying 5	0.65	10.85
Workplace Bullying 6 Workplace Bullying 7 Workplace Bullying 8 Workplace Bullying 9	0.751 0.768 0.624 0.654	8.342 5.645 8.691 6.390
**Turnover intention** (AVE 0.66=, CR = 0.50,α = 0.78)		
Turnover Intension 1	0.75	Fixed
Turnover Intension 2	0.77	6.04
Turnover Intension 3	0.69	8.08
**Creative performance** (AVE = 0.78, CR = 0.5, α = 0.88)		
Creative performance 1	0.77	8.836
Creative performance 2	0.68	7.625
Creative performance 3	0.74	6.844
Creative performance 4	0.87	8.946
Creative performance 5	0.584	Fixed
Creative performance 6	0.74	8.362

(X ²=359, df = 137, X2 /df = 2.62; CFI= 0.983; PNFI = 0.85; RMSEA = 0.060)

Table 3 provides an overview of the descriptive statistics and Pearson correlation coefficients among the primary variables examined in the study: narcissistic leadership, workplace bullying, turnover intention, and creative performance. This table outlines the average scores and standard deviations for each variable, as well as the direction and strength of their relationships. It serves as a foundational summary to better understand how these constructs are associated within the research framework.

**Table 3:** Descriptive statistics and Pearson correlations among study variables

	Mean	SD	1	2	3	4			
Narcissistic Leadership	3.2	0. 87	1						
Workplace Bullying	2.87	0.69	0.572	1					
Turnover Intension	3.02	0.74	0.277	0.289	1				
Creative performance	2.82	0.62	-0.400	-0.417	-0.266	1			

Note: Correlations are significant (*p* < .01, one-tailed test)

Skewness was used to verify that the data was normal. Leadership characterized by narcissism (0.907), bullying in the workplace (-1.131), intention to leave (1.027), and performance in terms of creativity (0.435) were the results. According to Lee and Yom [62] these results did not show any signs of non-normality.

Afterwards, a comparison was made between the fully mediated (x2 = 534.07, df = 187) and partially mediated (x2 = 493.51, df = 185). It appears that the fully mediated model is not as well-fitting as the hypothesized model (Δx² = 40.56, Λdf = 2, *p* < .01). The results for the hypothesis test were thus reported from the partially mediated model that provided a reasonable fit to the data (x2 = 493.51, df = 185, x2/df = 2.66; CFI = 0.94; PNFI = 0.76; RMSEA = 0.077; SRMR = 0.065). Because narcissistic leadership has a significant positive effect on workplace bullying (β = 0.681, t = 9.58), the empirical data lend credence to hypothesis 1.

Results show that H2a and H2b are supported by the data. The sentence means that narcissistic leadership influences turnover intention positively (β = 0.51, t = 8.37), but negatively influences creative performance (β = -0.436, t = -7.07).

Evidence from the experiments backs up hypotheses H3a and H3b, according to the results, bullying in the workplace has a positive influence on the intention to leave (β = 0.35, t = 5.48), but a negative impact on creative performance (β = -0.22, t = -2.39).

The Sobel test found that narcissistic leadership, through bullying in the workplace, affects both turnover intention (z = 3.29) and creative performance (z = -4.73). Bullying in the workplace is the linking mechanism of the relation between narcissistic leadership and both intention to leave and creative performance in the workplace. Bullying in the workplace, therefore, plays a mediating role in this process. Thus, Hypothesis 4 can be supported by empirical evidence.

**Fig. 2:**
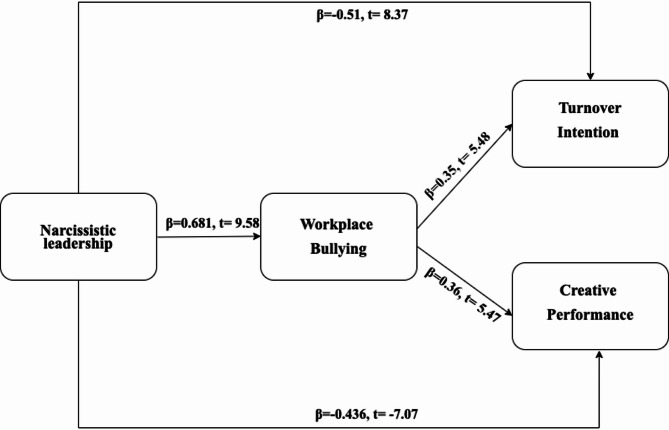
Hypothesized model results. x² =493.51, df = 185 478.41, x²/ df = 266; CFI = 0.94; PNFI = 0.76; RMSEA = 0.077; SRMR = 0.065

Table 4 Presents the standardized path coefficients, *t*-values, and 95% confidence intervals for the structural model tested in this study. The results indicate that narcissistic leadership is significantly and positively associated with workplace bullying (β = 0.681, *t* = 9.58, 95% CI [0.57, 0.79], *p* < .001), suggesting that higher levels of narcissistic leadership correspond to increased instances of bullying behavior in the workplace. Additionally, narcissistic leadership has a strong positive effect on turnover intention (β = 0.51, *t* = 8.37, 95% CI [0.42, 0.61], *p* < .001), and a significant negative effect on creative performance (β = − 0.436, *t* = − 7.07, 95% CI [–0.53, − 0.34], *p* < .001). Furthermore, workplace bullying positively predicts turnover intention (β = 0.35, *t* = 5.48, 95% CI [0.23, 0.46], *p* < .001) and negatively affects creative performance (β = − 0.36, *t* = − 5.47, 95% CI [–0.48, − 0.24], *p* < .001). All hypothesized relationships were statistically significant, providing strong empirical support for the proposed mediation model. These findings underscore the critical role of workplace bullying as a mechanism through which narcissistic leadership impacts key organizational outcomes (Fig. 2).

**Table 4:** Standardized path coefficients, t-values, and 95% confidence intervals for the structural model

Path	Standardized Coefficient (β)	t-value	95% Confidence Interval	Significance
Narcissistic Leadership → Workplace Bullying	0.681	9.58	[0.57, 0.79]	*p* < .001
Narcissistic Leadership → Turnover Intention	0.51	8.37	[0.42, 0.61]	*p* < .001
Narcissistic Leadership → Creative Performance	–0.436	–7.07	[–0.53, − 0.34]	*p* < .001
Workplace Bullying → Turnover Intention	0.35	5.48	[0.23, 0.46]	*p* < .001
Workplace Bullying → Creative Performance	–0.36	–5.47	[–0.48, − 0.24]	*p* < .001

## General discussion

An investigation into the role of bullying in the workplace as a mediator of the relationship between narcissistic leadership and nurses turnover intention and creative performance was the subject of our proposal and subsequent testing. Data was collected from nurses working in private hospitals in the city of Sulaimaniyah, which is located in the Kurdistan region of Iraq. All of the associations that were discussed earlier are given additional support by the findings that emerged from structural equation modeling. Bullying in the workplace can occur when there are negative leaders and managers present. These individuals have the ability to influence their followers to engage in unethical behavior and to create negative interpersonal relationships with them.

The Job Demands-Resources (JD-R) model suggests that when nurses are subjected to high job demands—such as working under a negative or unsupportive leadership style—they may experience strain that can lead to counterproductive behaviors such as workplace bullying (refer to 35). Moreover, both earlier and more recent research provides theoretical and empirical evidence supporting the link between narcissistic leadership and bullying in professional settings [36].

Narcissistic leadership may pose potential risks to organizational functioning. Not surprisingly, companies operating in different service settings (e.g., airlines, coffeehouses, hotels, restaurants) are well aware of several negative outcomes (e.g., employee grievance, procrastination, lower job performance) of narcissistic leadership.

Moroever, Using COR theory as a theoretical basis for the influence of workplace bullying on nurses’ outcomes [17, 46]. It has been suggested that workplace bullying can lead to an increase in turnover and the elimination of creative performance. According to the findings, workplace bullying acts as the link between narcissistic leadership and propensity, turnover tension, and creative performance. The presence of narcissistic leadership practices sends strong warnings to nurses that leaders and managers create a toxic work environment. Bullying at work can result in negative outcomes like turnover intentions and decreased creative performance of nurses.

### Theoretical implications

There are a number of theoretical findings that contribute to and enhance the existing body of knowledge regarding bullying in the workplace as a mediator of the effect of narcissistic leadership on nurses’ intention to leave their jobs and their creative performance.

To be more specific, our research investigates the aforementioned relationships by means of COR and JDR theories, and then puts these theories to the test by utilizing data collected from nurses working in private hospitals. According to recent research, it is essential to determine the underlying mechanism that links narcissistic leadership to the attitudes and behaviors of nurses based on strong theoretical foundations (for example, [49]). This is in agreement with the findings of the aforementioned research.

According to Xiao et al. [61] the majority of the studies that have been conducted on narcissistic leadership have focused on organizational identification. Therefore, it is important to treat bullying in the workplace as a mediator in this process.In addition, our research goes beyond the conventional method of determining whether or not an individual intends to leave their current position and investigates the tendency to leave as a result of bullying in the workplace. Empirical studies typically center their attention on the problem of nurses turnover. According to Anasori et al. and Holm et al. [17] bullying in the workplace is a factor that contributes to nurses turnover. Additionally, it discourages other nurses, which hinders their productivity and creativity [45]. Therefore, it is essential to have a thorough understanding of bullying in the workplace.

Last but not least, this investigation offers an alternative viewpoint by analyzing these phenomena within the framework of a developing economy, more specifically the Kurdistan region of Iraq. In the literature on hospitality management, there is a lack of empirical research on the perceptions of nurses regarding organizational practices and the potential consequences of narcissistic leadership in emerging economies. The incorporation of data from this one-of-a-kind setting contributes to the enhancement of the existing body of literature by providing a fresh viewpoint on how to interpret these phenomena. The findings of the study are significant because they highlight the universal significance of leadership dynamics, as well as the possibility of contextual variations in the effects of these dynamics. This study makes a contribution to the larger theoretical discourse on leadership by drawing attention to the fact that research on leadership would be incomplete without taking into account cultural and contextual factors.

### Implications on business practice

This research provides recommendations for managers on how to implement narcissistic leadership, workplace bullying, and the realization of negative outcomes. First, Fostering an environment that encourages nurses to voice any apprehensions they may possess pertaining to leadership conduct or instances of bullying at work. Establish mechanisms for anonymous feedback to guarantee the inclusion of all perspectives.

Second, Establishing unambiguous policies and protocols to tackle workplace bullying, encompassing reporting systems and repercussions for offenders. Provide training to managers and employees on identifying and resolving instances of bullying.

Third, Implementing comprehensive training initiatives aimed at equipping leaders with the necessary skills in effective communication, emotional intelligence, and empathy. Assist individuals in comprehending the ramifications of their conduct on both nurses and the overall organization.

Fourth, Cultivating an environment that values cooperation and teamwork as a means to mitigate the adverse consequences associated with narcissistic leadership. Promote collaboration among nurses to collectively pursue shared objectives and commemorate shared accomplishments.

Fifth, Establishing rewards and recognition systems to encourage nurses to contribute unique ideas and embrace creative risks. Acknowledge and incentivize individuals who make constructive contributions to the creative performance of the organization.

Finally, Monitorring staff morale and well-being by doing periodic check-ins, administering surveys, and organizing feedback sessions. Promptly address any indications of unhappiness or dissatisfaction to avert adverse consequences, such as elevated turnover rates.

### Limitations and directions for future research

The potential for bias in self-reported data is one of the constraints on the research on narcissistic leadership in private hospitals. This is because workers may be reluctant to talk about their experiences with bullying on the job or their thoughts about leaving the company. Response bias may also arise from social desirability, where participants provide answers they believe are more acceptable rather than reflecting their true experiences. Additionally, particular hospital settings or cultural contexts may impact the restrictions in generalizability, therefore it’s necessary to be cautious when applying the results to a larger population. Since the study was conducted within a single region and sector (private hospitals in Kurdistan), the findings may not fully capture the dynamics present in public healthcare settings or in other geographic contexts. Moreover, the cross-sectional research design limits the ability to establish causal relationships between narcissistic leadership, workplace bullying, turnover intention, and creative performance. Future studies may consider longitudinal or experimental approaches to better assess causality.

It is possible that future studies in this area will examine how narcissistic leadership affects private hospitals’ bottom lines, employee happiness on the job, and staff health. To mitigate the negative impact of narcissistic leadership on workplace dynamics, future studies can investigate the role of company culture and leadership development programs.

Furthermore, academic research might look into how narcissistic leadership affects creative output in the setting of private hospitals. Psychological safety, autonomy, and intrinsic drive are just a few of the many potential areas of investigation. Research into strategies or methods that develop an environment that is more supportive of creativity under narcissistic leadership could be a valuable direction for academic investigation in the future.

## Data Availability

The current study, which focuses on nurses, is non-sensitive, without collecting personal or sensitive data. All participants provided informed consent prior to filling the questionnaire. Ensured that participation was voluntary and that the data collected were kept anonymous, complying with standard ethical practices for such research. The datasets generated during and/or analyzed during the current study are available from the corresponding author upon reasonable request.

## Declarations

### Questionnaire/ scale

The current study depended on scales from previous empirical studies to measure the study variables:

### Narcissistic leadership

Hochwarter, W.A., andThompson, K.W. (2012). Mirror, mirroronmyboss’swall: engaged enactment’s moderating role on the relationship between perceived narcissistic supervision and work outcomes. Hum. Relate. 65, 335–366. Doi: 10.1177/0018726711430003.

### Workplace bullying

Einarsen, S. L., & Skogstad, A. (1996). Bullying at Work: Epidemiological Findings in Public and Private Organizations. European Journal of Work & Organizational Psychology, 5, 185–201. 10.1080/13594329608414854.

### Turnover intention

Singh, J., Verbeke, W. and Rhoads, G.K. (1996), “Do organizational practices matter in role stress processes? a study of direct and moderating effects for marketing oriented boundary spanners”, Journal of Marketing, Vol. 60 No. 3, pp. 6986.

### Creative performance

Wang, Guangping, and Richard G. Netemeyer. 2004. Salesperson creative performance: Conceptualization, measurement, and nomological validity. Journal of Business Research 57 (8): 805 − 12. 10.1016/S0148 2963(2)00483-6.

## References

[1] Wu H, Liu Y. The relationship between organisational support for career development, organisational commitment, and turnover intentions among healthcare workers in Township hospitals of henan, China. BMC Prim Care. 2022;23(1):136. 10.1186/s12875-022-01753-4. 35655133 10.1186/s12875-022-01753-4 PMC9161467

[2] Kaynak İ, Akıllıbaş E, Çiçek B. The role of narcissistic leadership in the effect of internal marketing on creative employee performance. Bus Manag Stud Int J. 2022;10(2):664–82. 10.15295/bmij.v10i2.2014.

[3] Park E, You CH, Joung H, Kwon YD. Effect of COVID-19 response work experience on turnover intention among employees of dedicated COVID-19 hospitals in Seoul. Hum Resour Health. 2024;22(1):39. 38872223 10.1186/s12960-024-00926-9 PMC11170911

[4] Islam MS, Amin M. A systematic review of human capital and employee well-being: putting human capital back on the track. Eur J Train Dev. 2022;46(5–6):504–34.

[5] Islam MS, Amin M, Feranita F, Karatepe OM. High-involvement work practices, work engagement and their effects on bank employees’ turnover intentions: the moderating role of functional competence. Int J Bank Mark. 2023;41(6):1360–88.

[6] Gruda D, McCleskey J, Karanatsiou D, Vakali A. I’m simply the best, better than all the rest: narcissistic leaders and corporate fundraising success. Pers Individ Dif. 2021;168:110317. 10.1016/j.paid.2020.110317.

[7] Aboramadan M, Turkmenoglu MA, Dahleez KA, Cicek B. Narcissistic leadership and behavioral cynicism in the hotel industry: the role of employee silence and negative workplace gossip. Int J Contemp Hosp Manag. 2020;33(2):428–47. 10.1108/IJCHM-04-2020-0315.

[8] Alhasnawi HH, Abbas AA. Narcissistic leadership and workplace deviance: a moderated mediation model of organizational aggression and workplace hostility. Organizacija. 2021;54(4):334–49. 10.2478/orga-2021-0019.

[9] Norouzinik Y, Rahimnia F, Maharati Y, Eslami G. Narcissistic leadership and employees’ innovative behaviour: mediating roles of job embeddedness and job engagement. Innov. 2022;24(3):355–80. 10.1080/14479338.2021.1917000.

[10] Wang H, Jiao R, Li F. Research on the effect of narcissistic leadership on employee job embeddedness. Front Psychol. 2022;13:927529. 10.3389/fpsyg.2022.927529. 35874331 10.3389/fpsyg.2022.927529 PMC9301298

[11] Asrar-ul-Haq M, Anjum T. Impact of narcissistic leadership on employee work outcomes in the banking sector of Pakistan. Futur Bus J. 2020;6(1):1–9. 10.1186/s43093-020-00014-6.

[12] Sakkar Sudha K, Shahnawaz MG. Grandiose narcissism and performance in organizations: mediating role of subjective well being. Leadersh Educ Personal Interdiscip J. 2020;2:101–11.

[13] Glad B. Why tyrants go too far: malignant narcissism and absolute power. Polit Psychol. 2002;23(1):1–37.

[14] Einarsen SL, Skogstad A. Bullying at work: epidemiological findings in public and private organizations. Eur J Work Organ Psychol. 1996;5:185–201.

[15] Al Muala I, Al-Ghalabi RR, Alsheikh GAA, Hamdan KB, Alnawafleh EAT. Evaluating the effect of organizational justice on turnover intention in the public hospitals of jordan: Mediated-Moderated model of employee silence, workplace bullying, and work stress. Int J Prof Bus Rev. 2022;7(3):3. 10.26668/businessreview/2022.v7i3.0820.

[16] Mendiratta A, Srivastava S. Workplace bullying and organizational citizenship behavior: the parallel mediating effects of job satisfaction and resilience. Int J Emerg Mark. 2023;18(7):1565–86. 10.1108/IJOEM-07-2021-1105.

[17] Holm K, Jönsson S, Muhonen T. How are witnessed workplace bullying and bystander roles related to perceived care quality, work engagement, and turnover intentions in the healthcare sector? A longitudinal study. Int J Nurs Stud. 2023;138:104429. 10.1016/j.ijnurstu.2023.104429. 36577260 10.1016/j.ijnurstu.2022.104429

[18] Malik MS, Sattar S. Declining employee engagement & employee performance: the noxious effects of workplace bullying. J Bus Soc Rev Emerg Econ. 2020;6(1):165–76.

[19] Keyes-Pretlow C. The correlation between workplace bullying, leader narcissistic tendencies, and the organizational culture within the financial sector [dissertation]. Grand Canyon University. 2021.

[20] Jang SJ, Lee H. Pathological narcissism, interpersonal cognitive distortions, and workplace bullying among nurses: a cross-sectional study. J Nurs Manag. 2022;30(7):3051–9. 10.1111/jonm.13698. 35688446 10.1111/jonm.13706

[21] Khan HSUD, Cristofaro M, Chughtai MS, Baiocco S. Understanding the psychology of workplace bullies: the impact of dark tetrad and how to mitigate it. Manag Res Rev. 2023;46(12):1748–68. 10.1108/MRR-04-2022-0256.

[22] Kim TT, Karatepe OM, Lee G, Lee S, Hur K, Xijing C. Does hotel employees’ quality of work life mediate the effect of psychological capital on job outcomes? Int J Contemp Hosp Manag. 2017;29(6):1638–57. 10.1108/IJCHM-04-2016-0224.

[23] Erkutlu HV, Chafra J. Leader narcissism and subordinate embeddedness: the moderating roles of moral attentiveness and behavioral integrity. EuroMed J Bus. 2017;12(2):189–202. 10.1108/EMJB-01-2016-0001.

[24] Yao Z, Zhang X, Liu Z, Zhang L, Luo J. Narcissistic leadership and voice behavior: the role of job stress, traditionality, and trust in leaders. Chin Manag Stud. 2020;14(3):543–63. 10.1108/CMS-12-2018-0878.

[25] Ghislieri C, Cortese CG, Molino M, Gatti P. The relationships of meaningful work and narcissistic leadership with nurses’ job satisfaction. J Nurs Manag. 2019;27(8):1691–9. 10.1111/jonm.12859. 31479543 10.1111/jonm.12859

[26] Alessandri G, Consiglio C, Luthans F, Borgogni L. Testing a dynamic model of the impact of psychological capital on work engagement and job performance. Career Dev Int. 2018;23(1):33–47. 10.1108/CDI-11-2016-0210.

[27] Saeed M. Mediation effect of psychological contract between personality dimensions and turnover intention. J Econ Finance Adm Sci. 2020;25(50):205–20. 10.1108/JEFAS-07-2019-0113.

[28] Karatepe OM, Ozturk A, Kim TT. Servant leadership, organisational trust, and bank employee outcomes. Serv Ind J. 2019;39(2):86–108. 10.1080/02642069.2018.1458157.

[29] Khan H, Rehmat M, Butt TH, Farooqi S, Asim J. Impact of transformational leadership on work performance, burnout and social loafing: A mediation model. Futur Bus J. 2020;6(1):1–13. 10.1186/s43093-020-00027-1.

[30] Freire C, Bettencourt C. Impact of ethical leadership on job satisfaction: the mediating effect of work–family conflict. Leadersh Organ Dev J. 2020;41(2):319–30. 10.1108/LODJ-07-2019-0325.

[31] Durrah O, Kahwaji A. Chameleon leadership and innovative behavior in the health sector: the mediating role of job security. Empl Responsibilities Rights J. 2023;35(2):247–65. 10.1007/s10672-023-09397-0. 10.1007/s10672-022-09414-5 PMC9203145 40477989

[32] Muzee H. Strategic leadership and employee engagement, evidences from an African industrial setting. Open Access Libr J. 2016;3(08):1. 10.4236/oalib.1102892.

[33] Hobfoll SE. Conservation of resources: a new attempt at conceptualizing stress. Am Psychol. 1989;44(3):513–24. 2648906 10.1037//0003-066x.44.3.513

[34] Karatepe OM, Rezapouraghdam H, Hassannia R. Job insecurity, work engagement and their effects on hotel employees’ non-green and nonattendance behaviors. Int J Hosp Manag. 2020;87:102472. 10.1016/j.ijhm.2020.102472.

[35] Hobfoll SE. The influence of culture, community, and the nested-self in the stress process: advancing conservation of resources theory. Appl Psychol Int Rev. 2001;50(3):337–421.

[36] Jaffar ZA, Mahdi MS, Hadi HMAA. Bullying at the workplace as a mediating variable between narcissistic leadership and organizational cynicism: an exploratory study in a selected sample in Kufa cement factory. J Posit Sch Psychol. 2022;6(8):5398–412.

[37] Ampofo ET, Karatepe OM. The effects of on-the-job embeddedness and its sub-dimensions on small-sized hotel employees’ organizational commitment, work engagement, and turnover intentions. Int J Contemp Hosp Manag. 2022;34(2):509–33. 10.1108/IJCHM-05-2021-0589.

[38] Hobfoll SE, Halbesleben J, Neveu JP, Westman M. Conservation of resources in the organizational context: the reality of resources and their consequences. Annu Rev Organ Psychol Organ Behav. 2018;5(1):103–28. 10.1146/annurev-orgpsych-032117-104640.

[39] Louis L. Leader’s perception of organizational narcissism density (OND), turnover intentions (TI), and the mediating role of organizational commitment (OC) [dissertation]. Alliant International University. 2015.

[40] Disque N. Influence of perceived supervisor support and narcissistic leadership on employee turnover intention [dissertation]. Walden University. 2020.

[41] Badar K, Aboramadan M, Plimmer G. Despotic vs narcissistic leadership: differences in their relationship to emotional exhaustion and turnover intentions. Int J Confl Manag. 2023;34(4):818–37. 10.1108/IJCMA-04-2022-0061.

[42] Zhou L, Li J, Liu Y, Tian F, Zhang X, Qin W. Exploring the relationship between leader narcissism and team creativity: evidence from R&D teams in Chinese high-technology enterprises. Leadersh Organ Dev J. 2019;40(8):916–31. 10.1108/LODJ-12-2018-0459.

[43] Liu X, Zheng X, Zhang Y, Liao H, Harms PD, Qin X, et al. Paradoxical effects of narcissism on creative performance: roles of leader–follower narcissism (in) congruence and follower identification with the leader. Hum Relat. 2024;77(1):3–31. 10.1177/00187267211025198.

[44] Lee RT, Ashforth BE. A meta-analytic examination of the correlates of the three dimensions of job burnout. J Appl Psychol. 1996;81(2):123–33. 10.1037/0021-9010.81.2.123. 8603909 10.1037/0021-9010.81.2.123

[45] Anasori E, De Vita G, Küçükergin GK. Workplace bullying, psychological distress, job performance and employee creativity: the moderating effect of psychological resilience. Serv Ind J. 2023;43(5–6):336–57. 10.1080/02642069.2022.2147514.

[46] Baek GL, Lee E. Impact of workplace bullying and resilience on new nurses’ turnover intention in tertiary hospitals. Nurs Health Sci. 2022;24(4):801–10. 10.1111/nhs.12968. 36096475 10.1111/nhs.12981

[47] De Clercq D, Fatima T, Jahanzeb S. Bullying and turnover intentions: how creative employees overcome perceptions of dysfunctional organizational politics. Pers Rev. 2022;51(9):2239–60. 10.1108/PR-10-2020-0792.

[48] Karatepe OM, Okumus F, Saydam MB. Outcomes of job insecurity among hotel employees during COVID-19. Int Hosp Rev. 2022; (ahead-of-print). 10.1108/IHR-09-2021-0064

[49] Olsen E, Bjaalid G, Mikkelsen A. Work climate and the mediating role of workplace bullying related to job performance, job satisfaction, and work ability: a study among hospital nurses. J Adv Nurs. 2017;73(11):2709–19. 10.1111/jan.13337. 28512986 10.1111/jan.13337

[50] Hochwarter WA, Thompson KW. Mirror, mirror on my boss’s wall: engaged enactment’s moderating role on the relationship between perceived narcissistic supervision and work outcomes. Hum Relat. 2012;65(3):335–66. 10.1177/0018726711430003.

[51] Einarsen S, Hoel H, Notelaers G. Measuring exposure to bullying and harassment at work: validity, factor structure and psychometric properties of the negative acts Questionnaire-Revised. Work Stress. 2009;23(1):24–44. 10.1080/02678370902815673.

[52] Coetzee M, Oosthuizen RM. Work-role psychosocial flourishing: its mediation role on workplace bullying and employee turnover intention. J Psychol Afr. 2017;27(3):211–5. 10.1080/14330237.2017.1321847.

[53] Hayat A, Afshari L. Supportive organizational climate: A moderated mediation model of workplace bullying and employee well-being. Pers Rev. 2021;50(7/8):1685–704. 10.1108/PR-06-2020-0455.

[54] Singh J, Verbeke W, Rhoads GK. Do organizational practices matter in role stress processes? A study of direct and moderating effects for marketing-oriented boundary spanners. J Mark. 1996;60(3):69–86. 10.2307/1251842.

[55] Wang G, Netemeyer RG. Salesperson creative performance: conceptualization, measurement, and Nomological validity. J Bus Res. 2004;57(8):805–12. 10.1016/S0148-2963(02)00483-6.

[56] Karatepe OM. Job resources, work engagement, and hotel employee outcomes: a time-lagged analysis. Econ Res Ekon Istraživanja. 2012;25(3):644–65. 10.1080/1331677X.2012.11517524.

[57] Anderson JC, Gerbing DW. Structural equation modeling in practice: a review and recommended two-step approach. Psychol Bull. 1988;103(3):411–23. 10.1037/0033-2909.103.3.411.

[58] Bagozzi RP, Yi Y. On the evaluation of structural equation models. J Acad Mark Sci. 1988;16(1):74–94. 10.1007/BF02723327.

[59] Fornell C, Larcker DF. Evaluating structural equation models with unobservable variables and measurement error. J Mark Res. 1981;18(1):39–50. 10.1177/002224378101800104.

[60] Soper T. Knowledge into learning: comparing lecture, e-learning and self-study take-home packet instructional methodologies with nurses. Nurs Open. 2017;4(2):76–83. 10.1002/nop2.73. 28286663 10.1002/nop2.73 PMC5340166

[61] Xiao X, Liu F, Zhou F, Chen S. Narcissistic leadership and employees’ knowledge sharing: influence of organizational identification and collectivism. Soc Behav Personal. 2018;46(8):1317–29. 10.2224/sbp.7034.

[62] Lee GS, Yom YH. Structural equation modeling on life-world integration in people with severe burns. Asian Nurs Res. 2013;7(3):112–9. 10.1016/j.anr.2013.07.002. 10.1016/j.anr.2013.06.003 25030248

